# Effect of Praziquantel on Preventing Delayed Infection of *Schistosoma japonicum* in Buffaloes and Goats

**DOI:** 10.3390/microorganisms13010017

**Published:** 2024-12-25

**Authors:** Xiang Gui, Bing Shao, Haoran Zhong, Rongxue Lv, Hao Li, Ke Lu, Yang Hong, Zhiqiang Fu, Zhenjie Lu, Mengge Xu, Yamei Jin, Jinming Liu

**Affiliations:** 1 National Reference Laboratory for Animal Schistosomiasis, Key Laboratory of Animal Parasitology of Ministry of Agriculture and Rural Affairs, Shanghai Veterinary Research Institute, Chinese Academy of Agricultural Sciences, Shanghai 200241, China; grayson0222@163.com (X.G.); pray1111111@163.com (B.S.); haoranzhong@shvri.ac.cn (H.Z.); Lvrongxue1227@163.com (R.L.); lihao@shvri.ac.cn (H.L.); luke@shvri.ac.cn (K.L.); hongyang_7@126.com (Y.H.); fuzhiqiang@shvri.ac.cn (Z.F.); 17701817707@163.com (Z.L.); 15290423703@163.com (M.X.); yameijin@shvri.ac.cn (Y.J.); 2 National Institute of Parasitic Diseases, Chinese Center for Diseases Control and Prevention (Chinese Center for Tropical Diseases Research), National Health Commission of the People’s Republic of China (NHC) Key Laboratory of Parasite and Vector Biology, World Health Organization (WHO) Collaborating Center for Tropical Diseases, National Center for International Research on Tropical Diseases, Shanghai 200025, China

**Keywords:** *Schistosoma japonicum*, praziquantel, livestock, prevention

## Abstract

Schistosomiasis, caused by *Schistosoma japonicum*, continues to pose a major public health threat in East Asia, with an estimated 71 million people at risk of infection. Domestic animals, especially buffaloes and goats, serve as important reservoir hosts, facilitating the transmission of the parasite to humans. While praziquantel (PZQ) is the first-line treatment for schistosomiasis due to its broad-spectrum efficacy against adult schistosomes, its prophylactic potential is less explored. This study aimed to evaluate the efficacy of PZQ in preventing *S. japonicum* infection in buffaloes and goats via assessing worm burden, worm size, hematological changes, and immune modulation. In the present study, buffaloes and goats were pretreated with PZQ at various doses (7–25 mg/kg body weight), followed by infection with *S. japonicum* cercariae. The results showed significant reductions in total worm burden and female worm burden, with one oral administration at 13 mg/kg for buffaloes and one injection at 25 mg/kg for goats offering the most robust protection. Worm length was also significantly reduced in both buffaloes and goats, indicating that PZQ not only prevented infection in this study but also inhibited worm growth. Furthermore, PZQ pretreatment modulated immune responses, as evidenced by increased levels of nitric oxide (NO) and interleukin-2 (IL-2) in buffaloes and Lym% in goats. These findings suggest that PZQ has significant prophylactic potential in livestock, offering a practical solution for reducing schistosome transmission from animals to humans in endemic regions. Additionally, this study indicates that PZQ pretreatment does not contribute to resistance development, as newly established infections are effectively cleared during the initial treatment window.

## 1. Introduction

Schistosomiasis, particularly caused by *Schistosoma japonicum*, remains a major public health issue in East Asia, where approximately 71 million human individuals are at risk of infection [1]. Domestic animals, especially buffaloes and goats, serve as significant reservoir hosts, facilitating the transmission of *S. japonicum* to humans in endemic regions [2,3]. Effective control of this zoonotic transmission cycle is essential for reducing the burden of schistosomiasis [4].

Praziquantel (PZQ) is widely regarded as the most effective treatment for schistosomiasis due to its broad-spectrum activity against adult schistosomes [5]. PZQ’s mechanism of action primarily involves the disruption of calcium ion homeostasis in the parasite. PZQ binds to voltage-gated calcium channels located on the parasite’s tegument, inducing a rapid influx of calcium ions. This results in sustained muscular contraction, paralysis, and eventual tegumental disruption, facilitating the host immune system’s ability to eliminate the parasite [6]. However, PZQ has a short elimination half-life, approximately 2.85–5.75 h in humans, 6.24 h in goats, and 7.73 h in cattle, which limits its duration of protection [7,8]. This suggests that a single dose may not sustain a long-term blood concentration sufficient for extended prophylaxis, and livestock can still become reinfected with *S. japonicum* after drug metabolism.

In addition to its direct action on schistosomes, PZQ has been reported to modulate the host immune response. It enhances the expression of certain cytokines, such as interferon-gamma (IFN-γ) and interleukin-2 (IL-2), which may augment the host’s ability to eliminate parasites [9,10]. Thus, it raises the question of whether pre-administration of PZQ prior to exposure in schistosome-endemic environments could provide temporary resistance through immune modulation. Previous studies by our group demonstrated that pretreatment with PZQ (two doses separated by 24 h via oral administration at 200 mg/kg body weight or a single intramuscular injection at 200 mg/kg body weight) protected mice against *S. japonicum* infection for 18 days [10]. These findings suggest that PZQ may induce immune-physiological changes that could temporarily reduce *S. japonicum* infection in livestock, even after complete drug metabolism.

The present study aims to determine the effective prophylactic dose of PZQ in preventing *S. japonicum* infection in buffaloes and goats. By establishing a preventative dosing strategy, this research seeks to provide a practical solution for reducing schistosome transmission from livestock to humans, thereby contributing to ongoing schistosomiasis control efforts in endemic regions.

## 2. Materials and Methods

### 2.1. Ethics Statement

The animal experiment protocol used in this study was approved by the Institutional Animal Care and Use Committee at the Shanghai Veterinary Research Institute in China (Permit No: SHVRI-SZ-20200427-01). All procedures administered to the animals in this study followed the guidelines of the Association for Assessment and Accreditation of Laboratory Animal Care International.

### 2.2. Animals, Parasites, and Drugs

Buffaloes (male, 340–450 kg, 1–2 years old) and goats (male, 30–40 kg, 2–3 months old) were sourced from a schistosomiasis-free region in Nantong, Jiangsu Province, China. Two weeks prior to the experiment, all animals were weighed and treated with ivermectin to eliminate potential gastrointestinal nematode infections. The *S. japonicum* Anhui strain was maintained in our laboratory with cercariae obtained by exposing infected snails to light. PZQ was acquired from Hubei Aibo Technology Co., Ltd. (Xianning, China) and the PZQ solution was prepared in a saturated borneol solution, as previously described [10].

### 2.3. Animal Grouping, PZQ Pretreatment, and Challenge Infection

In the buffalo trial, nine animals were stratified by live weight into two pretreatment groups (13 mg/kg and 25 mg/kg) and one control group, with three buffaloes in each group. In the goat trial, thirty-two goats were similarly stratified by live weight into three pretreatment groups (7 mg/kg, 13 mg/kg, and 25 mg/kg) and one control group, with eight goats in each group. All buffaloes and goats in the pretreatment groups were weighed on day −16 and then pretreated with a single dose of PZQ on day −15. Buffaloes in the pretreatment groups were administered oral doses, with a maximum dose of 10 g, while those in the control group remained untreated. For goats, injection was chosen over oral administration as it is more convenient and efficient for determining effective dosage. Therefore, goats were injected with PZQ dissolved in saturated borneol solution at doses ranging from 7 to 25 mg/kg body weight (BW). Goats in the control group received an equivalent amount of saturated borneol solution.

On day 0, all animals were percutaneously infected with *S. japonicum* cercariae (approximately 500 cercariae for buffaloes, 300 cercariae for goats). The animals were sacrificed on days 54–56 for worm recovery and liver sample collection. Blood samples were collected from the jugular veins of buffaloes and goats on days −16, −1, 14, and 54–56. The PZQ pretreatment and challenge protocol are presented in Supplementary Figure S1.

### 2.4. Efficacy Assessment

Worms were obtained through hepatic portal perfusion [11]. All parasites recovered from each animal were counted. Liver tissue samples (an approximately 1 g liver tissue sample of each animal) were processed, and eggs were collected and counted using established methods, as described in previous studies [10]. The number of eggs per gram (EPG) of liver tissue was calculated for each animal [12]. Reduction in the worm burden, female worm burden, and liver egg count exceeding 50% was considered the threshold for an effective protective dose.

### 2.5. Worm Morphology Observation

After the worm counting of each animal, the recovered worms from different animals in each group were rinsed with PBS, pooled, and fixed with 10% formalin solution (Servicebio, Wuhan, China). Ten pairs of worms (10 males and 10 females) were randomly selected from each group and were fixed in FAA solution, and slide specimens were prepared using previously described methods [13]. The specimens were then examined under a microscope (Nikon, Tokoy, Japan). If fewer than 10 pairs of worms were recovered, all available paired worms were observed. The length of the worms from both experiments was measured. In the goat experiment, the length and width of the oral sucker and ventral sucker of both male and female worms were recorded, along with the length and width of the ovary in female worms, as well as the number of eggs within the uterus.

### 2.6. Hematology Analysis and Cytokine and NO Assays

Whole blood samples (2 mL) were analyzed with a Mindray BC5300 hematology analyzer (Mindray, Shenzhen, China). The parameters measured were the following: white blood cells (WBCs), lymphocytes (Lym), neutrophils (Neu), monocytes (Mon), basophils (Bas), and eosinophils (Eos).

Serum levels of buffalo IL-2, IFN-γ, and IL-10 and goat IL-2, IFN-γ, and TNF-α were determined using enzyme-linked immunosorbent assay (ELISA) kits (Mlbio, Shanghai, China). Nitric oxide (NO) levels were measured by an NO assay kit (Dogesce, Beijing, China). All samples were tested in triplicate according to the manufacturer’s instructions.

### 2.7. Statistical Analysis

Data were analyzed using SPSS software version 20.0. (SPSS Inc., Chicago, IL, USA). The Kolmogorov–Smirnov test was used to validate the normality of distribution. Data are presented as the mean ± standard deviation (x ± s). A one-tailed *t*-test was used to compare differences between two groups; one-way ANOVA was used to compare differences among multiple groups. All *p*-values < 0.05 were taken as statistically significant.

## 3. Results

### 3.1. Pretreatment with PZQ Reduced the Worm Burden and Egg Count

**Figure 1:**
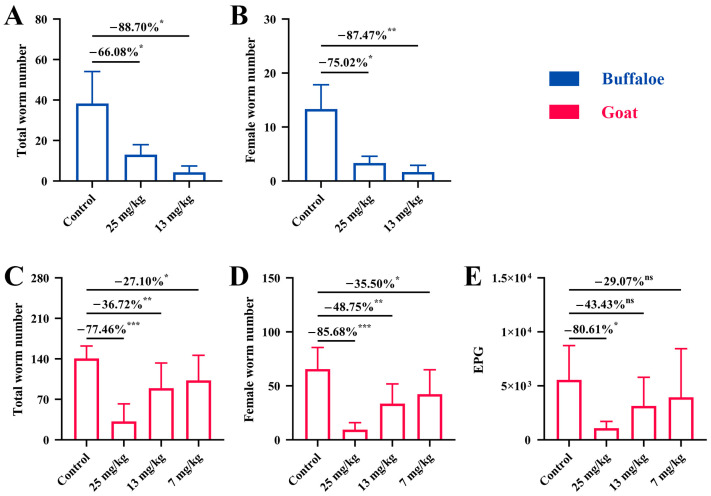
Effect of PZQ pretreatment on worm burden and egg production in buffaloes and goats. (**A**) Total worm burden in buffaloes after pretreatment with PZQ at doses of 25 mg/kg and 13 mg/kg BW. (**B**) Female worm burden in buffaloes following PZQ pretreatment at 25 mg/kg and 13 mg/kg BW. (**C**) Total worm burden in goats pretreated with PZQ at 25 mg/kg, 13 mg/kg, and 7 mg/kg BW. (**D**) Female worm burden in goats after PZQ pretreatment at 25 mg/kg, 13 mg/kg, and 7 mg/kg BW. (**E**) Egg per gram (EPG) count of liver tissue in goats, with reductions across PZQ treatment groups. Significant reductions in worm burden and egg counts are indicated by ns: not significant, * *p* < 0.05, ** *p* < 0.01, and *** *p* < 0.001. Data are presented as the mean ± SD.

As illustrated in Figure 1, both the total worm burden and female worm burden were significantly lower in the buffalo groups treated with 25 mg/kg and 13 mg/kg of PZQ compared to the control group. Specifically, the total worm burden in the 25 mg/kg and 13 mg/kg groups decreased by 66.08% (*p* < 0.05) and 88.70% (*p* < 0.05), respectively (Figure 1A). Similarly, the female worm burden in these groups showed reductions of 75.02% (*p* < 0.05) and 87.47% (*p* < 0.01), respectively (Figure 1B). There was no statistically significant difference between the 25 mg/kg and 13 mg/kg groups, indicating no clear dose-dependent effect. Additionally, due to the low number of worms recovered in the buffalo trial, it was difficult to accumulate sufficient liver eggs for detection. Collectively, these results confirmed that the efficacy of a single oral administration of 13 mg/kg BW in preventing *S. japonicum* infection in buffaloes.

In the goat trial, all PZQ-pretreated groups demonstrated lower values for all measured indices compared to the control group (Figure 1C–E). A single injection of PZQ at 25 mg/kg BW significantly reduced the total worm burden by 77.46% (*p* < 0.001), female worm burden by 85.68% (*p* < 0.001), and liver egg count by 80.61% (*p* < 0.05). The reductions in total worm burden and female worm burden for the 13 mg/kg and 7 mg/kg groups were less pronounced, but still statistically significant at 36.72% (*p* < 0.01) and 27.10% (*p* < 0.05) for total worm burden, and 48.75% (*p* < 0.01) and 35.50% (*p* < 0.05) for female worm burden, respectively. However, no significant reduction was observed in the liver egg count for the 13 mg/kg and 7 mg/kg groups. These results highlight that a single injection of 25 mg/kg BW PZQ provided the most robust protection in goats, with significant reductions in worm burden, female worm burden, and liver egg count, demonstrating its efficacy in preventing *S. japonicum* infection.

### 3.2. Pretreatment with PZQ Reduced the Size of Worms

**Figure 2:**
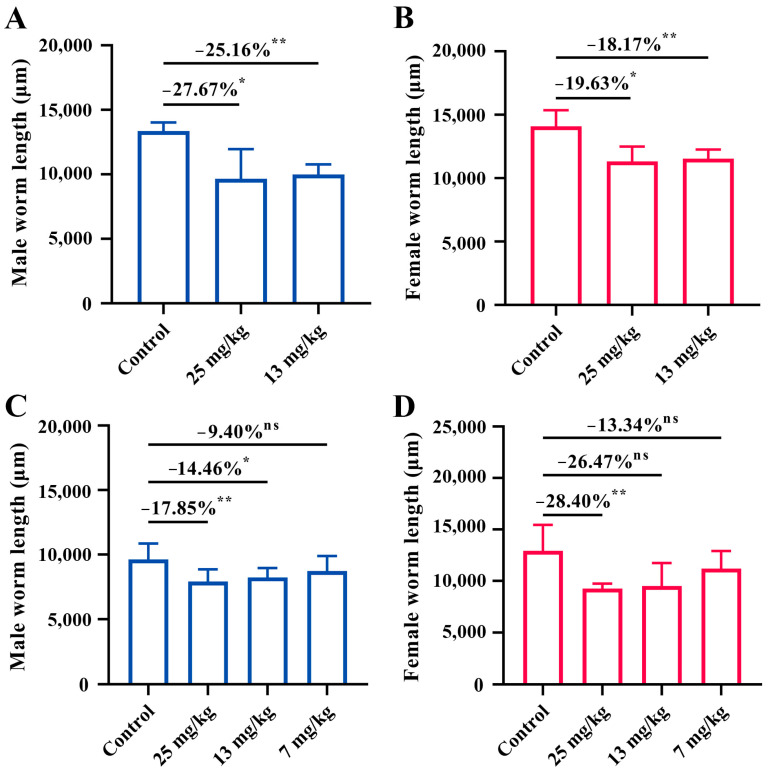
Impact of PZQ pretreatment on worm length in buffaloes and goats. (**A**) Male worm length in buffaloes after pretreatment with PZQ at doses of 25 mg/kg and 13 mg/kg BW. (**B**) Female worm length in buffaloes following PZQ pretreatment at 25 mg/kg and 13 mg/kg BW. (**C**) Male worm length in goats pretreated with PZQ at 25 mg/kg, 13 mg/kg, and 7 mg/kg BW. (**D**) Female worm length in goats following PZQ pretreatment at 25 mg/kg, 13 mg/kg, and 7 mg/kg BW. Statistical significance is indicated by ns: not significant, * *p* < 0.05, and ** *p* < 0.01. Data are presented as the mean ± SD.

As shown in Figure 2, pretreatment with PZQ resulted in a significant reduction in worm length for both male and female worms compared to the control group. In buffaloes, the average male worm length in the 25 mg/kg and 13 mg/kg groups was reduced by 27.67% (*p* < 0.05) and 25.16% (*p* < 0.01), respectively (Figure 2A). Similarly, the female worm length decreased by 19.63% (*p* < 0.05) and 18.17% (*p* < 0.01) in the 25 mg/kg and 13 mg/kg groups, respectively (Figure 2B). No significant dose-dependent effect was observed between these two dose groups, suggesting that both 25 mg/kg and 13 mg/kg effectively reduced worm length in buffaloes, leading to notable morphological changes.

In goats, PZQ pretreatment also significantly reduced worm length. The male worm length in the 25 mg/kg, 13 mg/kg, and 7 mg/kg groups decreased by 17.85% (*p* < 0.01), 14.46% (*p* < 0.05), and 9.40% (*p* > 0.05), respectively (Figure 2C). Female worms showed reductions of 28.40% (*p* < 0.01), 26.47% (*p* > 0.05), and 13.34% (*p* > 0.05) in the 25 mg/kg, 13 mg/kg, and 7 mg/kg groups, respectively (Figure 2D). Moreover, pretreatment with 25 mg/kg of PZQ resulted in the most substantial reductions in the length and width of the oral sucker and ventral sucker of both male and female worms, the length and width of the ovary in female worms, and the number of eggs within the uterus (Supplementary Table S1). Collectively, among the tested doses, the 25 mg/kg group consistently caused the greatest morphological changes in both male and female worm lengths in goats, suggesting that 25 mg/kg was more effective at reducing worm size compared to the 13 mg/kg and 7 mg/kg doses.

### 3.3. Hematological Changes

**Figure 3:**
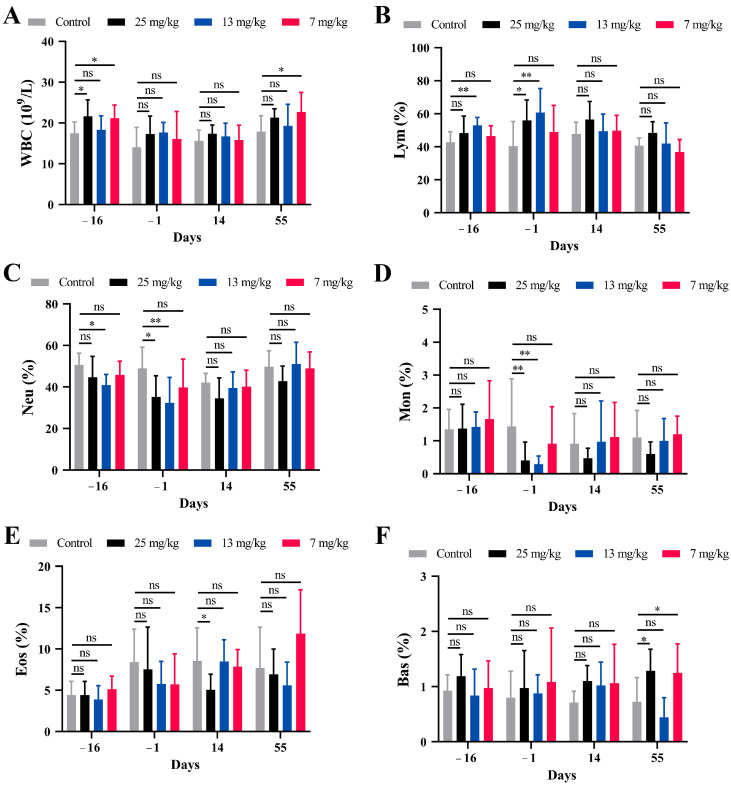
Changes in hematological parameters in goats following PZQ pretreatment. (**A**) White blood cell (WBC) counts. (**B**) Neutrophil (Neu) percentages. (**C**) Lymphocyte (Lym) percentages. (**D**) Basophil (Bas) percentages. (**E**) Eosinophil (Eos) percentages. (**F**) Monocyte (Mon) percentages. PZQ pretreatment was administered at doses of 25 mg/kg, 13 mg/kg, and 7 mg/kg BW, and blood samples were collected on days −16, −1, 14, and 55. Statistical significance is indicated by ns: not significant, * *p* < 0.05, and ** *p* < 0.01. Data are presented as the mean ± SD.

In the buffalo trial, no significant changes in hematological indicators were observed between the PZQ-treated and control groups. However, in the goat trial, PZQ pretreatment led to significant changes. The WBC significantly increased on day −16 in the 25 mg/kg and 7 mg/kg groups compared to the control group (Figure 3A). The Lym% was significantly elevated on day −1 in the 25 mg/kg and 13 mg/kg groups (Figure 3B). Conversely, Neu% and Mon% decreased significantly in the same groups on day −16 and day −1 (Figure 3C,D). Eos% in the 7 mg/kg group showed significant increases on day 55 (Figure 3E). Bas% exhibited less consistent trends, with significant changes observed only at specific time points or in certain groups (Figure 3F).

### 3.4. Serum Levels of Cytokines and NO

**Figure 4:**
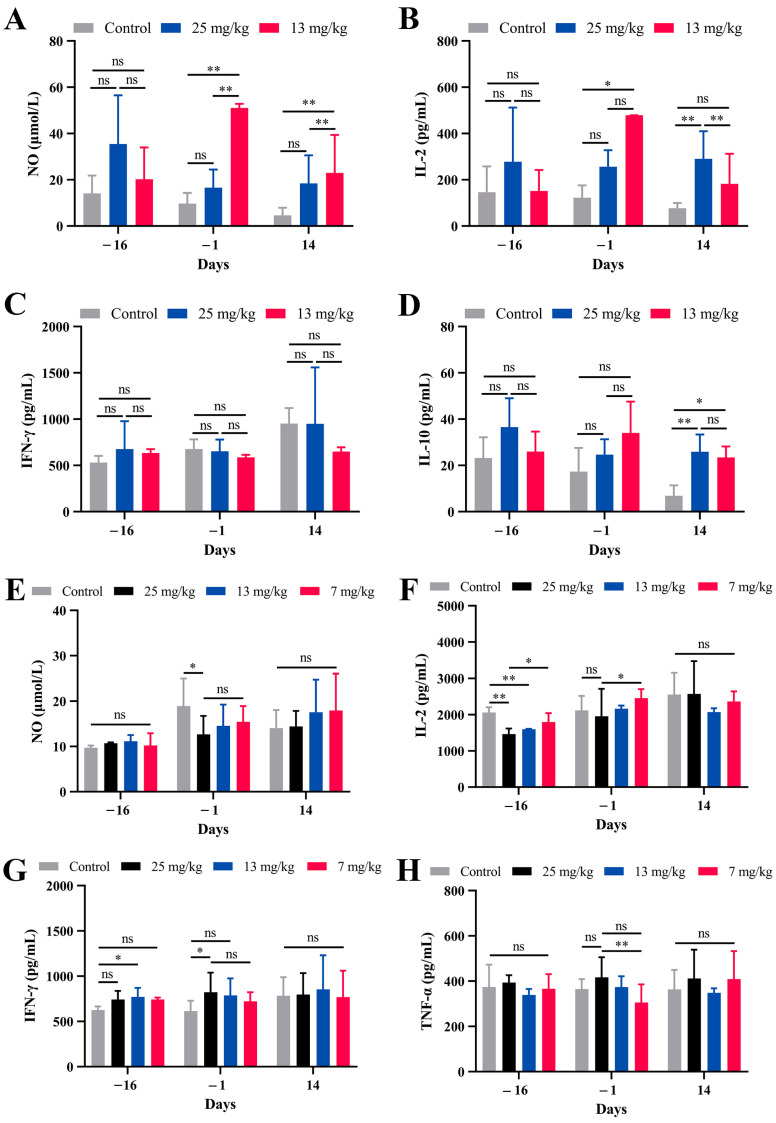
Serum levels of immune mediators in buffaloes and goats pretreated with PZQ. (**A**–**D**) Nitric oxide (NO), interleukin-2 (IL-2), interferon-gamma (IFN-γ), and interleukin-10 (IL-10) levels in buffaloes following PZQ pretreatment at 25 mg/kg and 13 mg/kg BW. (**E**–**H**) NO, IL-2, IFN-γ, and tumor necrosis factor alpha (TNF-α) levels in goats following PZQ pretreatment at 25 mg/kg, 13 mg/kg, and 7 mg/kg BW. Blood samples were collected on days −16, −1, and 14. Statistical significance is indicated by ns: not significant, * *p* < 0.05, and ** *p* < 0.01. Data are presented as the mean ± SD.

The serum levels of NO, IFN-γ, IL-10, IL-2, and TNF-α in each group of buffaloes and goats were detected at different time points. In buffaloes, NO levels significantly increased on day −1 and day 14 in the 13 mg/kg group compared to both the control group and the 25 mg/kg group (Figure 4A). IL-2 levels were also elevated on day −1 in the 13 mg/kg group (Figure 4B). However, there were no significant changes in IFN-γ or IL-10 levels at most time points in buffaloes (Figure 4C,D). In the goat trial, the levels of NO and IL-2 increased progressively with the duration of PZQ treatment, showing significant differences at certain time points (Figure 4E,F). The level of IFN-γ was significantly elevated in the 25 mg/kg group on day −1 compared to the control group (Figure 4G). In contrast, TNF-α levels showed no major differences between the groups (Figure 4H).

## 4. Discussion

Recent studies in the Philippines have reported a 97% infection rate in water buffaloes, with an average worm burden of 94 worms (95% confidence interval, 49–138 worms) [3]. Similarly, in the Lindu Subdistrict of Central Sulawesi Province, Indonesia, the overall prevalence of schistosomiasis in domestic animals was found to be 32.9%, with the highest rates in cattle (61.5%) and buffaloes (42.3%) [14]. These findings highlight the critical role that livestock plays in maintaining the transmission cycle of *S. japonicum*, contributing significantly to the transmission of schistosomiasis to humans in these regions.

While PZQ is the primary treatment for schistosomiasis, it does not prevent reinfection [15]. Notably, previous studies from our laboratory revealed that PZQ also possesses prophylactic potential [10]. Building on these findings, this study aimed to evaluate its efficacy as a preventive measure in buffaloes and goats. By assessing worm burden, worm size, hematological changes, and immune modulation, we sought to provide insights that could support the development of effective control strategies in regions with high *S. japonicum* prevalence.

In this study, PZQ pretreatment significantly reduced the worm burden in buffaloes and goats, with the greatest reductions observed in the 13 mg/kg group. These findings are consistent with previous reports of PZQ’s therapeutic efficacy [8]; however, our results demonstrate its potential as a prophylactic measure. Additionally, liver egg counts were markedly reduced in PZQ-pretreated goats, further corroborating the protective effect of PZQ. Notably, our study indicated that even at lower cercariae infection doses, as in our experiment (500 cercariae for buffaloes and 300 for goats), PZQ pretreatment remains highly effective, reducing worm burden by more than 80%. These results provide important reference data for implementing similar prophylactic strategies in highly endemic regions such as the Philippines and Indonesia.

Interestingly, we observed significant reductions in worm length across all PZQ-treated groups, particularly at 13 mg/kg in buffaloes and 25 mg/kg in goats. Moreover, in the 25 mg/kg group of goats, the worms exhibited significant reductions in the sizes of their oral sucker, ventral sucker, and ovary, along with a notable decrease in their number of uterine eggs, indicating a pronounced effect on worm morphology. These results suggest that PZQ not only prevents the establishment of infection but may also inhibit the growth of worms that survive the initial immune clearance. Previous studies have indicated that smaller worms contribute fewer eggs, which could reduce transmission to humans [16]. Therefore, in areas where animal-to-human transmission is a major concern, reducing worm size through PZQ pretreatment could play an important role in controlling the disease’s spread [17].

The changes in hematology, serum NO, and cytokines observed in this study provide further insights into the effects of PZQ pretreatment. In goats, PZQ pretreatment resulted in significant increases in Lym% and reductions in Neu% and Mon%, particularly in the 25 mg/kg or 13 mg/kg groups. Moreover, serum cytokine profiles revealed that PZQ pretreatment modulates immune responses by increasing NO and IL-2 levels in buffaloes and IFN-γ in goats of 25 mg/kg group (Figure 4). The PZQ pretreatment of mice can induce physiological changes, including higher levels of NO, IFN-γ, and IL-2 and lower levels of TGF-β [10]. NO is known to play a key role in immune-mediated schistosome killing [18], and its upregulation following PZQ treatment suggests a possible mechanism through which PZQ exerts its prophylactic effects. Similarly, elevated IL-2 levels may reflect enhanced T-cell proliferation and an improved immune response, which is critical for controlling schistosome infections [19].

One of the concerns surrounding the widespread use of PZQ is the potential for resistance development, particularly if the drug is used extensively for both treatment and prevention [20]. However, our study suggests that the risk of PZQ resistance development is minimal. As observed, PZQ was highly effective in clearing mature worms that infected the host before treatment, as well as worms that established themselves within 1–3 days after PZQ administration [10]. Once the drug was cleared from the bloodstream, any surviving worms or new infections were not exposed to subtherapeutic doses of PZQ, reducing the likelihood of resistance emergence. Moreover, no detectable drug residues were observed after the treatment window, further supporting that that PZQ pretreatment was unlikely to lead to long-term resistance.

Despite these promising findings, our study has several limitations. The sample size for buffaloes was relatively small, which may limit the generalizability of our findings in larger populations. Additionally, buffaloes exhibit a self-healing phenomenon in response to *S. japonicum* infection, leading to fewer worms being collected, and consequently, liver egg counts could not be reliably measured [21]. Future studies should include a larger number of animals to confirm the observed effects or consider increasing the infection dose in buffaloes to better assess the impact of PZQ. Moreover, the buffaloes and goats used in this study were not standardized laboratory animals, which may have contributed to greater variability between individuals. While this study suggests that PZQ induces immune modulation, particularly through the upregulation of NO and IL-2, the exact pathways through which PZQ confers protection against *S. japonicum* remain unclear. Further research is needed to elucidate the specific immune mechanisms involved and to identify potential molecular targets of PZQ. Additionally, the efficacy of PZQ against other *Schistosoma* species prevalent in Africa and South Asia remains unexplored and warrants investigation [14].

In conclusion, this study provides compelling evidence that PZQ is effective not only as a treatment for infected animals but also as a prophylactic agent in livestock. The reductions in worm burden, inhibition of worm growth, and modulation of immune parameters suggest that PZQ could play a critical role in schistosomiasis control programs targeting livestock reservoirs. While the delayed infection effect observed in this study may be regarded as a supplementary outcome compared to the WHO-recommended population-level preventive treatment, its practical relevance remains significant. By reducing initial infection rates and worm burdens in individual animals, this approach has the potential to complement existing strategies by targeting zoonotic reservoirs, thereby reducing transmission risks to humans. In this study, the optimal effective dose of PZQ was identified as 13 mg/kg administered orally in buffaloes and 25 mg/kg administered via injection in goats. Based on these results, we propose monthly PZQ administration at the therapeutic dose during the susceptible season for all ruminants that are often in constant contact with contaminated water. However, further research is required to validate these findings in larger animal populations and explore its efficacy against other *Schistosoma* species in highly endemic regions.

## Data Availability

The original contributions presented in this study are included in the article/supplementary material. Further inquiries can be directed to the corresponding author.

## Supplementary Materials

The following supporting information can be downloaded at: https://www.mdpi.com/article/10.3390/microorganisms13010017/s1, Figure S1: Schedule of pretreatment, blood sample collection, challenge, and sacrifice; Table S1: Worm morphometry from goats pretreated with PZQ and controls.
