# Supplementary material for: Effect of Praziquantel on Preventing Delayed Infection of Schistosoma japonicum in Buffaloes and Goats
Source: Microorganisms. 2024 Dec 25;13(1):17. doi: 10.3390/microorganisms13010017 (PMC11768081; doi:10.3390/microorganisms13010017)
Supplement: Supplementary file 1 [file microorganisms-13-00017-s001.zip › Supplementary Table S1.pdf]

Supplementary Table S1 Worm morphometry from goats pretreated with PZQ and controls

Doses and Groups	Oral sucker (μm)		Ventral sucker (μm)		Ovary (μm)		Number of uterus eggs
	length	width	length	width	length	width	
Male worm							
Control	236.23±19.44	156.08±33.83	316.94±33.13	170.48±22.36	/	/	/
25mg/kg	194.23±20.36*	134.26±17.03	277.87±11.08*	157.64±13.86	/	/	/
13mg/kg	205.32±6.89*	139.13±8.60	278.12±4.91*	164.76±5.24	/	/	/
7mg/kg	216.13±13.87*	142.21±18.17	313.80±16.07	160.97±10.03	/	/	/
Female worm							
Control	51.32±5.41	34.51±2.95	58.71±0.74	38.58±1.03	656.23±86.85	192.07±15.27	36.33±6.18
25mg/kg	37.92±1.61*	29.68±2.99	51.68±2.89*	32.99±0.09	482.65±17.83*	143.64±6.89*	16.95±0.72*
13mg/kg	44.01±3.87	31.75±0.51	54.53±1.33*	31.54±0.58*	500.49±43.83*	156.48±10.70*	20.75±2.86*
7mg/kg	46.52±0.86	30.29±0.14*	55.33±1.64	36.47±0.51	546.94±46.69	177.85±17.74	19.75±7.98*

Data are presented as the mean ± standard deviation; length and width in μm; * $P < 0.05$, compared with control.
