# Supplementary figures and images for: Effect of Praziquantel on Preventing Delayed Infection of Schistosoma japonicum in Buffaloes and Goats
Source: Microorganisms. 2024 Dec 25;13(1):17. doi: 10.3390/microorganisms13010017 (PMC11768081; doi:10.3390/microorganisms13010017)

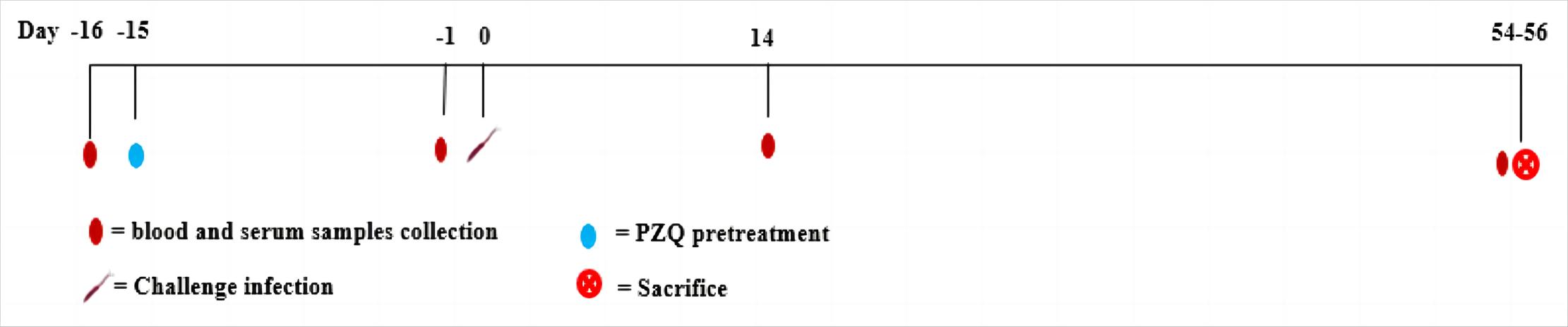

Supplement: Supplementary file 1 [file microorganisms-13-00017-s001.zip › Supplementary Figure S1.jpg]
